# Supplementary material for: Protein-protein interaction network controlling establishment and maintenance of switchable cell polarity
Source: PLoS Genet. 2020 Jun 22;16(6):e1008877. doi: 10.1371/journal.pgen.1008877 (PMC7332107; doi:10.1371/journal.pgen.1008877)
Supplement: S1 Text — (DOCX) [file pgen.1008877.s019.docx]

**Supplementary Appendix: Extended model discussion and analysis.**

**Protein-protein interaction network controlling establishment and maintenance of switchable cell polarity**

Luís António Menezes Carreira^1, 4^, Filipe Tostevin^1, 2, 4^, Ulrich Gerland^2^

& Lotte Søgaard-Andersen^1, 3^

^1^ Department of Ecophysiology, Max Planck Institute for Terrestrial Microbiology,

Karl-von-Frisch Str. 10,

35043 Marburg, Germany

^2^ Physik-Department, Technische Universität München,

James Franck Straße 1,

85747 Garching, Germany

^3^ Corresponding author

Tel. +49-(0)6421-178201

Fax +49-(0)6421-178209

E-mail: sogaard@mpi-marburg.mpg.de

^4^ These authors contributed equally

**Cytoplasmic diffusion**

The typical length of an *M. xanthus* cell is ~6 µm. Observations of GFP diffusion in bacteria under different conditions suggest that a diffusion coefficient of D~10 µm^2^/s is reasonable for a small cytoplasmic protein [1,2]. With these values, the mixing timescale over which cytoplasmic proteins can be expected to explore the bulk of the cell will be ~6^2^/(2×10)~1.8 s. The turnover rate of the polarity proteins in polar clusters was previously measured by FRAP [3], which identified the recovery timescale of MglA as 4 s, and longer for MglB and RomR. Thus, cytoplasmic diffusion is somewhat faster than the timescale of polar binding/unbinding, meaning that concentration gradients in the cytoplasm will dissipate on the timescale of polar turnover. It is therefore reasonable to approximate the cytoplasm as being well-mixed.

Furthermore, note that in steady-state there can be no net diffusive fluxes along the cell. Consequently, the mean protein concentration must be constant along the cell length. Therefore, the steady-state of a model incorporating one-dimensional diffusion along the long axis of the cell will be equivalent to that of a model with a well-mixed cytoplasm. This argument does not rule out the possibility of local gradients and circulatory fluxes once the three dimensional nature of the cytoplasm is taken into account. However, protein localization spots appear to be confined to the cell poles and do not extend to the lateral cell membrane. Additionally, the short axes of *M. xanthus* are ≲0.5 µm meaning that diffusive mixing across the short axis will be extremely fast. Given these observations, it seems implausible that significant transverse gradients could be maintained.

**Parameter estimation**

As noted in the Methods section, model parameters were determined through a combination of manual and automatic fitting to the experimentally-observed polar protein localization patterns (Table S5; Fig 5A-C). In the automatic fitting, the objective function was chosen to be the total squared deviation of the polar protein fractions between experiment and model, $\sum_{s\in strains} \sum_{X=A,B,R} \sum_{i=1,2} \left( X_{i,s,model}-\text{<}X_{i,s,exp}\text{>} \right)^{2}$, where the set of *X*_model_ values depend on the choice of model parameters. We attempted a fully-automated global parameter search using randomly-determined initial trial parameter values. However, we found that this optimization procedure failed to identify parameter combinations that generated bistability in the WT condition, instead becoming trapped in monostable regimes where the objective function changed only slowly with parameters. In contrast, we were readily able to manually identify parameter combinations for which the model WT localization pattern was qualitatively similar to the experimental observations. We therefore decided to use manual curation to initialize the automatic fitting. To this end, we manually varied the model parameters and visually compared the resulting model behavior to the experimental data, iterating this process to obtain initial trial parameter values that provided good qualitative and quantitative agreement between the model and experiment.

First, the polar dissociation rates for RomR and MglB, *d*_R_ and *d*_B_, were fixed according to the polar fluorescence recovery times measured in FRAP experiments [3]. For MglA, since GAP-induced dissociation is expected to play a significant role, the spontaneous dissociation rate *d*_A_ was chosen to be approximately half that predicted by the relocation timescale measured by FRAP. No such reduction was made for MglB so that (i) the chosen value was in accordance with experiments in the variant model where MglA does not act on MglB, and (ii) the low amounts of MglA at poles where MglB is present suggest that direct regulation of MglB by MglA is not the primary mechanism of polar MglB turnover.

For the remaining parameters we exploited the combinatorial nature of the different mutants to isolate individual or subsets of interactions where possible. With the dissociation rates fixed as above, we used the Δ*mglB*Δ*romR* double-mutant condition to determine the spontaneous association rate *k*_A_ by minimizing the difference between the (symmetric) state of the model and the experimentally observed mean total polar fractions, and similarly for *k*_B_ and the *ΔmglAΔromR* condition. For RomR, the polar localization pattern in the Δ*mglA*Δ*mglB* strain is insufficient to constrain both the binding rates *k*_R_ and *k*_RR_ and the bias in polar *R* localization, *R*_bias_. For this reason, we initially assumed a bias of 5% (*R*_bias_=0.05), and estimated the other parameters accordingly. During the later automatic parameter fitting, *R*_bias_ was again allowed to vary. Next, the behavior of the Δ*mglB* mutant was used to determine the recruitment of *A* by *R*, *k*_AR_, and the Δ*mglA* mutant was used to choose the mutual recruitment rates of *B* and *R*, *k*_BR_ and *k*_RB_. Since the polar fractions of both MglA and MglB in the Δ*romR* condition are extremely low and it is hard to clearly discern the effects of this mutation, we did not directly use this strain to determine the MglA-MglB interaction parameters *d*_AB_ and *d*_BA_. Instead, we simultaneously chose these parameters together with the MglA-RomR negative feedback strength *K*, by matching to the wild-type localization pattern.

The parameter set generated by the above procedure (except for the dissociation rates, *d*_A_, *d*_B_ and *d*_R_, which were held constant throughout) were then used to initialize the automated fitting. This was carried out by minimizing the total squared deviation between model and experiment, defined above, in log-parameter space using the Broyden-Fletcher-Goldfarb-Shanno algorithm [4] from the SciPy Python library [5]. This is a deterministic algorithm that uses only the Jacobian of the objective function and estimates the search direction via a quasi-Newton method. Repeated runs of the fitting procedure while varying initial parameters by ±10% produced similar optimal parameter sets, as did repeating the procedure with the output parameter set as the initial trial.

**Linear stability analysis of mutants**

In this section we use linear stability analysis to show that within our model, a symmetric (i.e. *A_1_*=*A*_2_, *B*_1_=*B*_2_, *R*_1_=*R*_2_) steady-state is stable in all depletion mutant conditions, and that this state becomes unstable only when all three polarity proteins are present. Since we are interested in the stability of the symmetric state, we will consider the model in the absence of any intrinsic bias in RomR localization. We will focus on the full model equations shown in Fig. 6B, incorporating both suppression of MglB-RomR recruitment by MglA and active displacement of MglB by MglA. Only in the case of all three proteins do qualitative differences emerge between the different variant models incorporating only one of these effects. For the full model (in any of its variants), stability analysis is not analytically tractable. Numerical investigation of the stability of these models is discussed below.

In the following it becomes convenient to change variables into the sum and difference of polar protein fractions, which we denote respectively as *X*_p_=*X*_1_+*X*_2_=1-*X*_c_ and Δ*X*=*X*_1_-*X*_2_, where *X*=*A*, *B* or *R*. We use *X** to denote the value of *X* evaluated at steady-state.

Δ*mglB*Δ*romR* and Δ*mglA*Δ*romR*

In the Δ*mglB*Δ*romR* double mutant, the steady-state equations for *A* reduce to


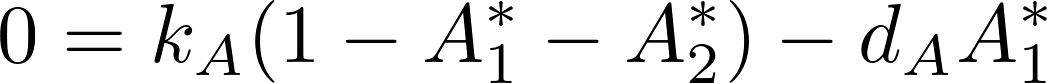
 (1)


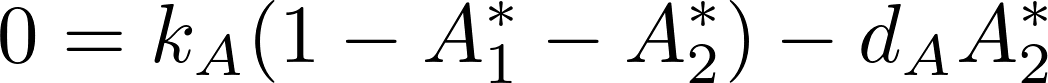
. (2)

These equations have a single solution,


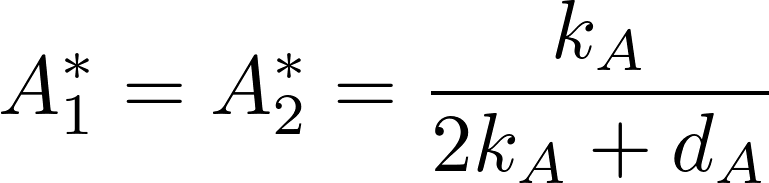
,

which is evidently symmetrical. Evaluating the Jacobian matrix


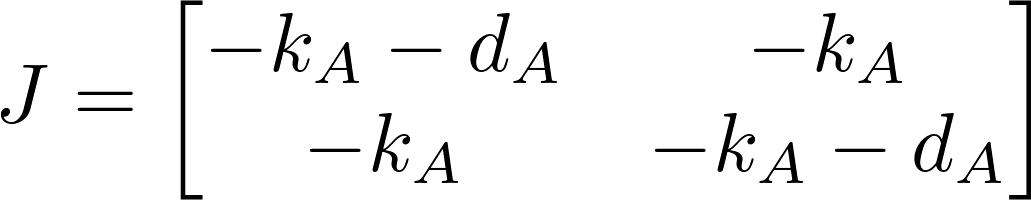
,

the corresponding eigenvalues are


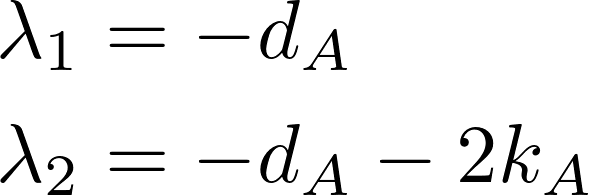
,

which are both negative. Thus the symmetric condition is stable.

The equations for *B* in the Δ*mglA*Δ*romR* double mutant are equivalent with *A* replaced by *B* throughout. Thus also in this scenario there is a single stable symmetric fixed point.

Δ*mglA*Δ*mglB*

In the Δ*mglA*Δ*mglB* double mutant, the steady-state equations for *R* reduce to


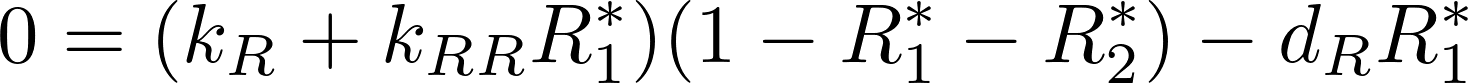
 (3)


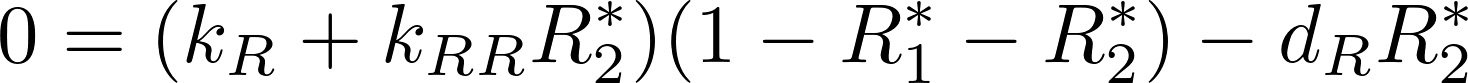
 (4)

Calculating the sum and differences of Eqs (3) and (4) and changing variables, we obtain


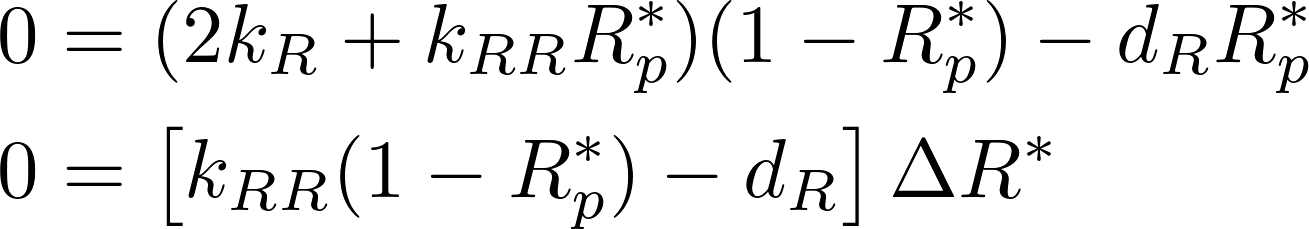
,

which have solutions Δ*R**=0, meaning that all steady-state solutions are symmetric, and


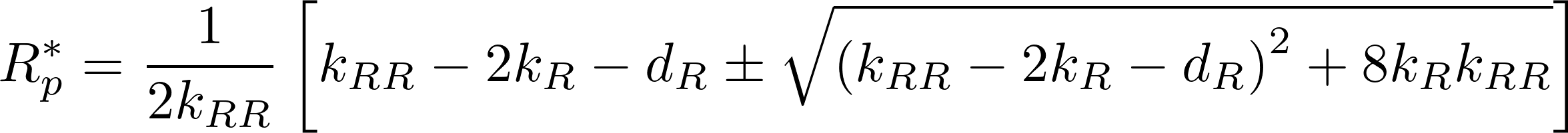
.

Of these solutions the positive root is always greater than zero and the negative root is always less than zero; therefore the positive root is the physically realistic one. The resulting Jacobian matrix is


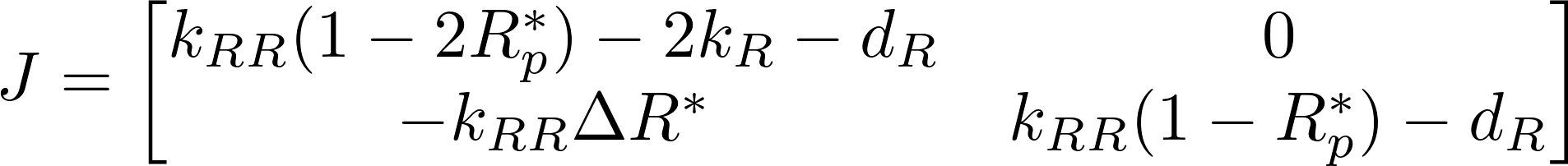
.

Evaluating the eigenvalues at the positive solution for *R*_p_*, we find


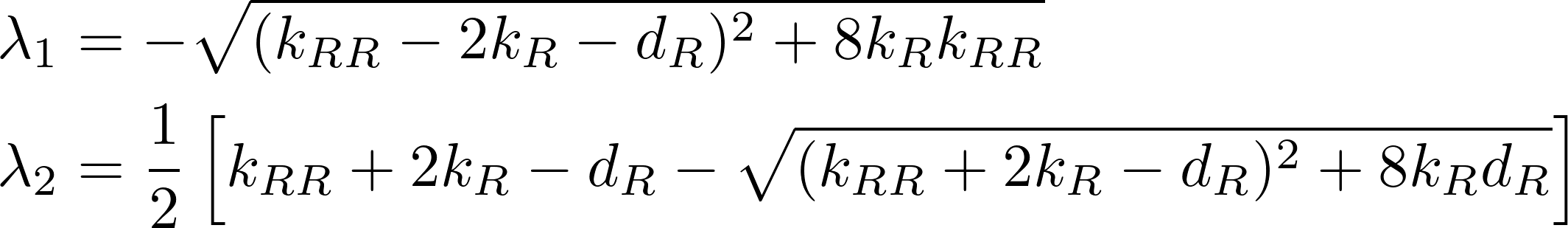
,

both of which are real and negative for all non-negative parameter values. Thus the symmetric solution is stable.

Δ*mglB*

In the absence of *B*, the steady-state equations for *A* and *R* become


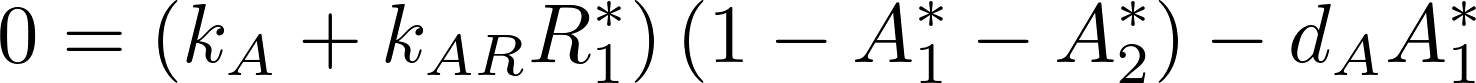
 (5)


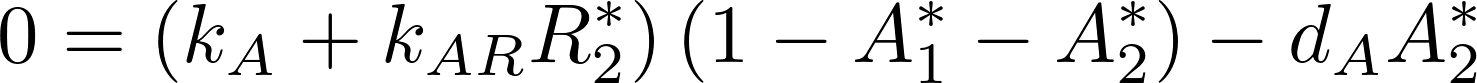
 (6)


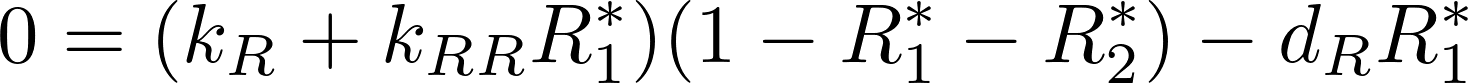
 (7)


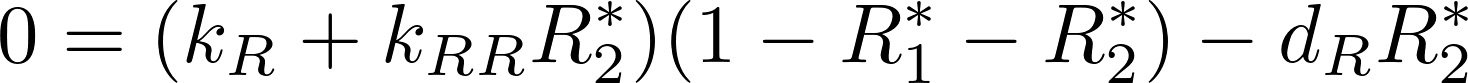
. (8)

Since *A* does not appear in the equations for *R*, Eqs (7) and (8), the solutions for these equations are as above for the Δ*mglA*Δ*mglB* scenario. These solutions can then be inserted into (5) and (6) to find the modified solutions for *A*. For brevity we will not present the full solutions here. However, it is straightforward to see that Eqs (5) and (6) imply that


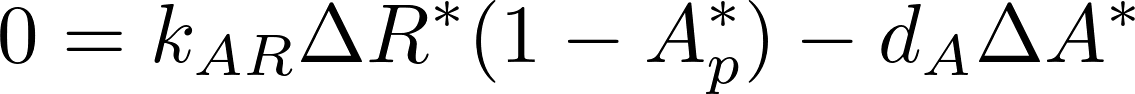
,

and since Δ*R**=0, it must follow that Δ*A**=0. Thus the only physically realistic steady-state of this mutant is also symmetrical. Additionally, two of the eigenvalues of the resulting Jacobian have the same form as in the Δ*mglA*Δ*mglB* scenario. The remaining two eigenvalues are


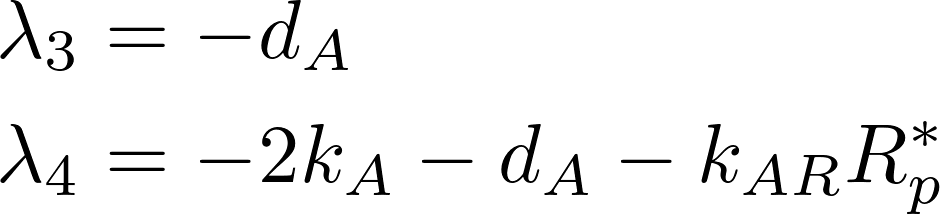
.

Since all four eigenvalues are strictly negative, the symmetric steady-state is again stable.

Δ*romR*

The situation in the absence of *R* is somewhat more involved than the cases considered thus far. Rather than presenting the solutions in full, we will limit our analysis to demonstrating that the model cannot generate a stable steady-state solution with *A* and *B* polarized in opposite directions, while any realistic symmetric steady-state solution must be stable. The steady-state equations for *A* and *B* in this scenario are


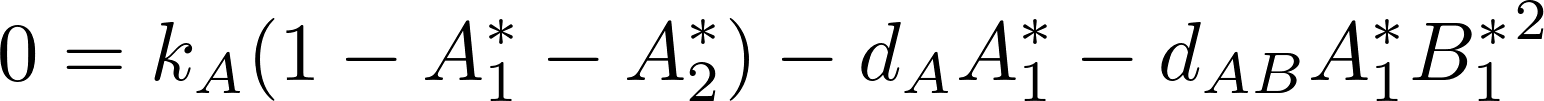
 (9)


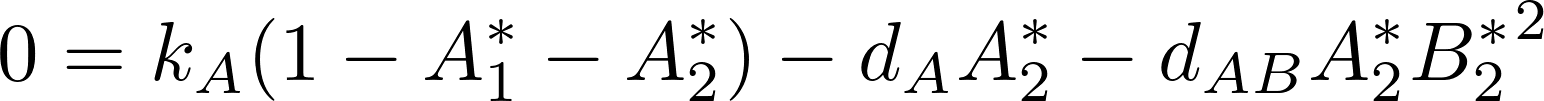
 (10)


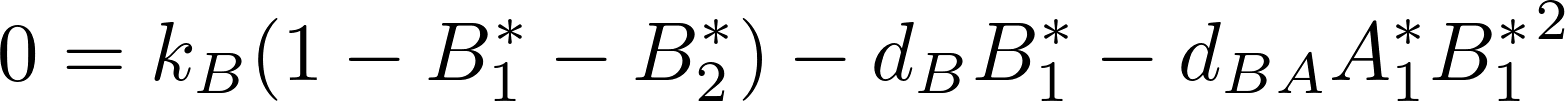
 (11)


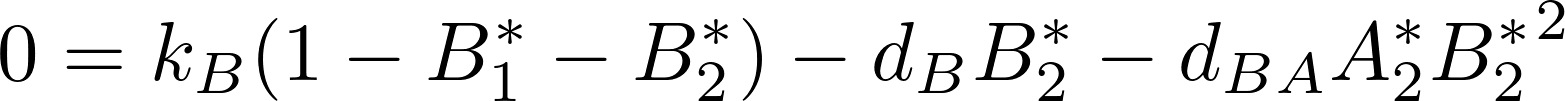
. (12)

Direct solution of these equations leads to nine distinct steady-state solutions, though it is not immediately clear which are physically realistic (i.e. values of *A*_1_, *A*_2_, *B*_1_ and *B*_2_ all lying in the allowable range [0,1]). Of these nine solutions, three have Δ*A*=*Δ*B**=0; the remaining six solutions all have the same sign for Δ*A** and Δ*B**. To see that Eqs (9-12) require this, we first eliminate the third-order terms by computing *d*_BA_×(9)-*d*_AB_×(11), and similarly for (10) and (12):


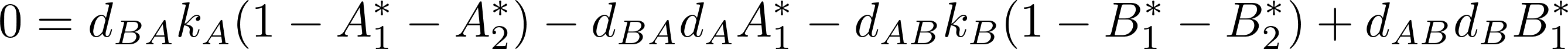
 (13)


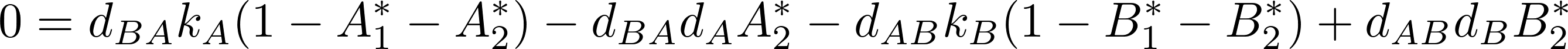
. (14)

Next we again take the difference Eqs (13) and (14), which yields


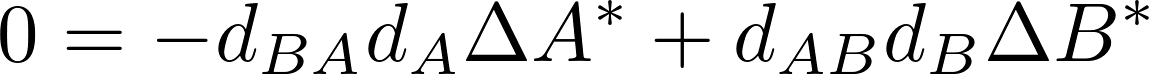
. (15)

Equation (15) implies that, if there is any steady-state asymmetry, then both *A* and *B* must be polarized in the same direction, since Δ*A** and Δ*B** must have the same sign.

Evaluating the Jacobian matrix (omitted for brevity) at the symmetric steady-state solutions, the resulting eigenvalues all have the form


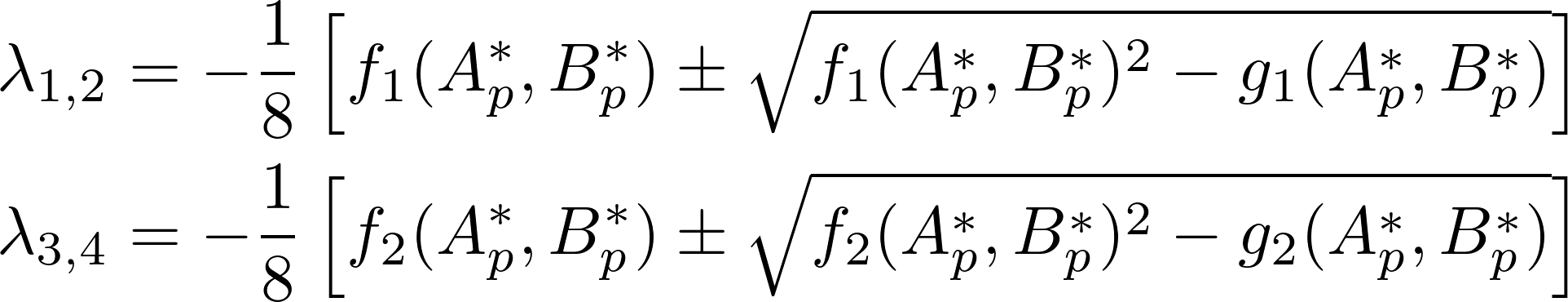


where the functions


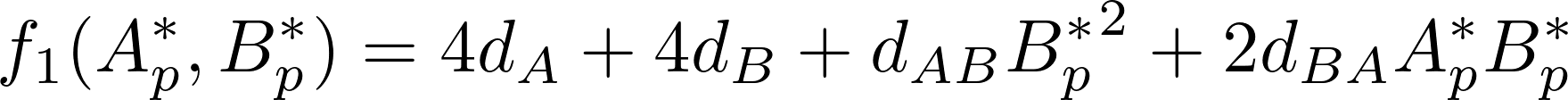


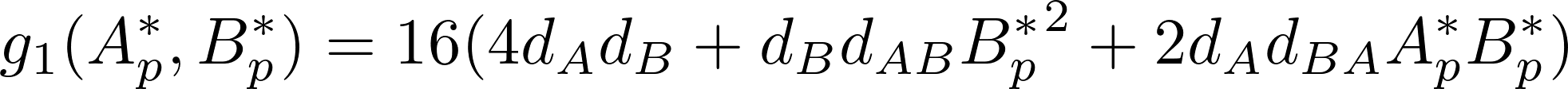


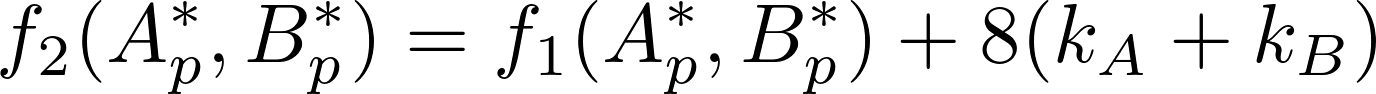


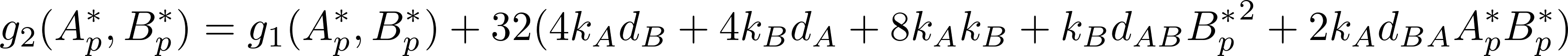


are strictly positive as long as *A*_p_* and *B*_p_* are positive. Consequently, any physically-realistic symmetric solution of Eqs (9-12) must also be stable, since the eigenvalues are negative if *g*_i_ ≤ *f*_i_^2^, or complex with negative real part if *g*_i_ > *f*_i_^2^. In any case, it is not possible for these equations to generate a steady state configuration in which *A* and *B* have opposite polarities.

Δ*mglA*

In the absence of *A*, the steady-state equations for *B* and *R* become


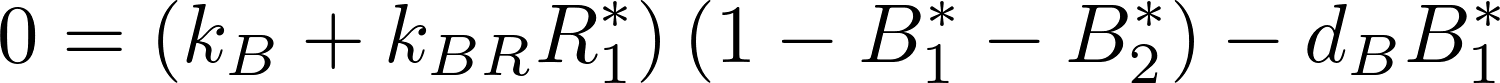
 (16)


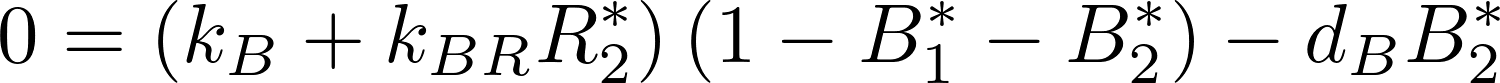
 (17)


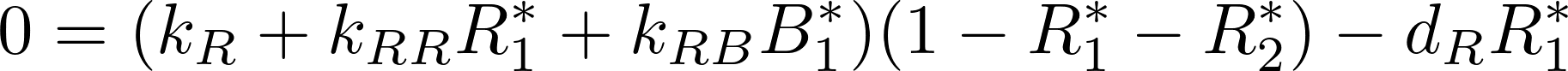
 (18)


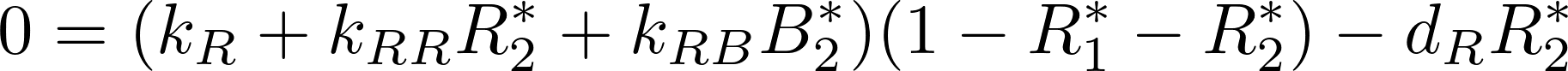
. (19)

Changing variables, we find


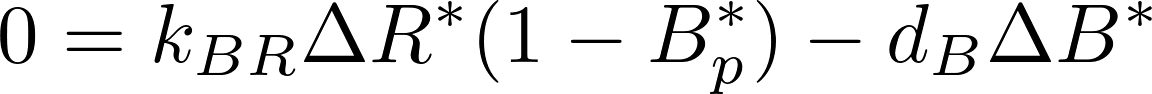
 (20)


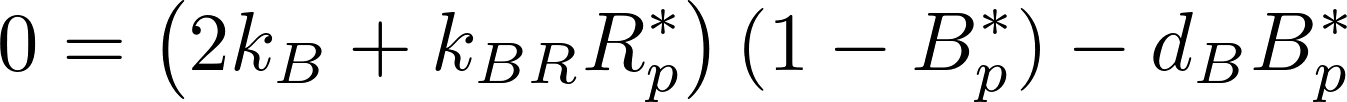
 (21)


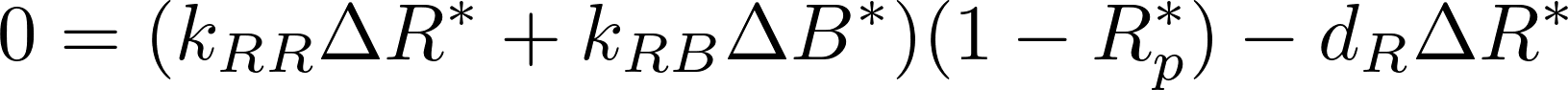
 (22)


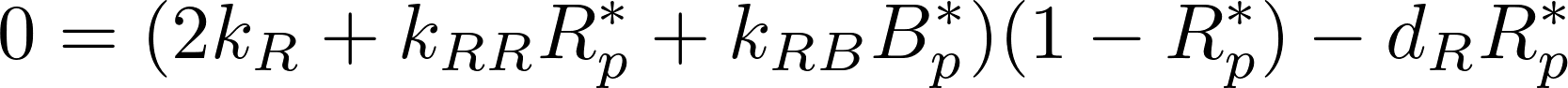
. (23)

Together, Eqs (20) and (22) require that Δ*B**=Δ*R**=0. In all there are three distinct steady-state solutions with different *B*_p_* and *R*_p_* (not given here in full), though two may be non-physical. Evaluating the Jacobian in (Δ*B*, *B*_p_, Δ*R*, *R*_p_) space, we find that the eigenvalues can again be written in the form


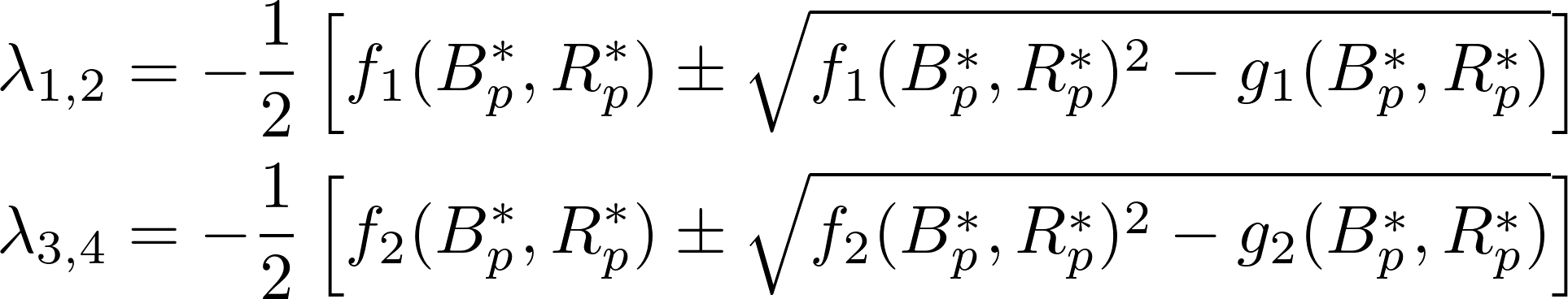


where


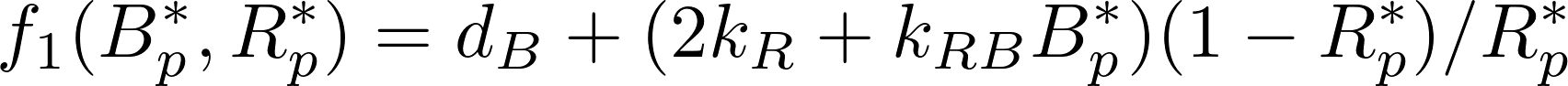


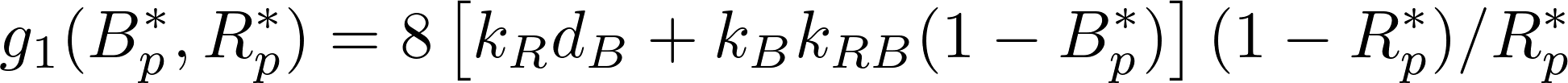


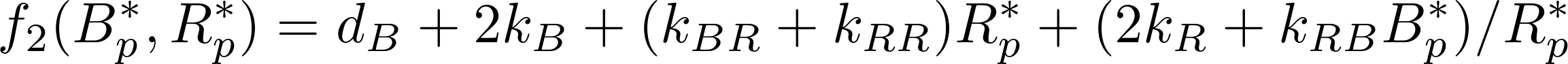


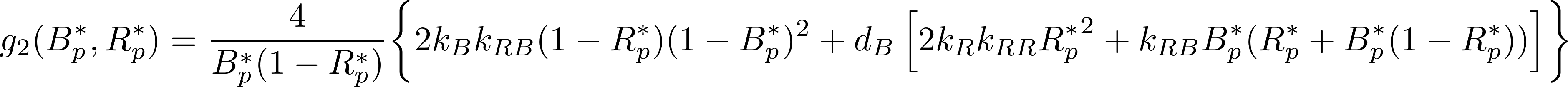


These functions are again strictly positive as long as 0<*B*_p_*<1 and 0<*R*_p_*<1, such that any physical fixed point must be stable.

**Stability analysis of variant models**

As noted above, stability analysis for the WT models including all three proteins is not analytically tractable. Instead, we investigated the existence of different stability regimes numerically.

I) Direct regulation of MglB by MglA

For the model with only direct regulation of MglB by MglA (Fig. 6A-C, orange), with the other model parameters determined as described above, we were unable to identify combinations of *d*_AB_ and *d*_BA_ that lead to localization patterns in which the WT configuration was asymmetric and in qualitative agreement with the experimentally-observed WT pattern, either manually or by fitting; when the automatic parameter optimization algorithm was initialized in this state, it produced only symmetric configurations. To test whether bistability was possible in this model we tested the behavior of a random sampling of parameter combinations when each parameter value was allowed to vary over the range [10^-3^,10^5^]. In this test we did observe polarization with the same protein polarities as observed experimentally. However, in these simulations MglA had a strong intrinsic affinity for the poles (i.e. independently of RomR, large *k*_A_). Subsequently, systematic parameter sweeps confirmed that the region of instability expands with increasing *k*_A_, and we were unable to observe instability for *k*_A_≲1 min^-1^ (see Fig. A1). In this parameter regime, MglA accumulates at the poles in large amounts, independently of RomR (see Fig. A2), which is inconsistent with our experimental observations that MglA polar localization is low in the absence of, and significantly enhanced by, RomR. For this reason, we conclude that this model is not able to generate spontaneous cell polarization in a parameter regime consistent with our experimental data.

**
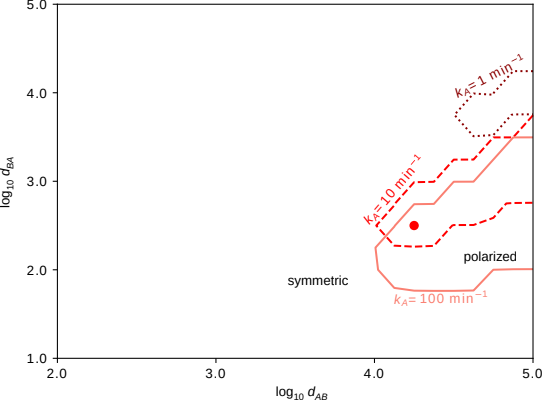
**

**Figure A1.** Estimated regions of instability in the *d*_AB_-*d*_BA_ plane. Lines show the estimated boundaries of the polarized regions for different values of the MglA polar recruitment rate *k*_A_*. For k*_A_ ≲1 min^-1^, no polarized region was observed. All other parameters were fixed at their values in Table S5. Point indicates the parameter combination shown in Fig. A2.


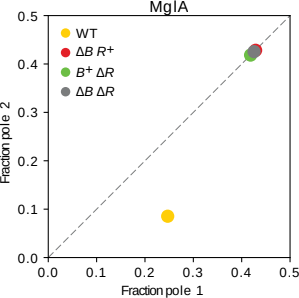
**Figure A2.** MglA polar fractions for large *k*_A_. Steady state polar fractions of MglA for the parameter values indicated by the point in Fig. A1 (*k*_A_=10 min^-1^, *d*_AB_=17780 min^-1^, *d*_BA_=316 min^-1^). Remaining parameters are as in Table S5.

II) Regulation of MglB-RomR feedback by MglA only

The qualitative behavior of this model is extremely similar to that of the full model, discussed in the main text. The observed regions of instability extended over a larger range of parameter values, but remained most sensitive to the choice of the MglA feedback strength K (see Fig. A3). This indicates that feedback from MglA onto the mutual recruitment of MglB and RomR is the principal mechanism at play in the full model and is primarily responsible for generating WT polar asymmetry.


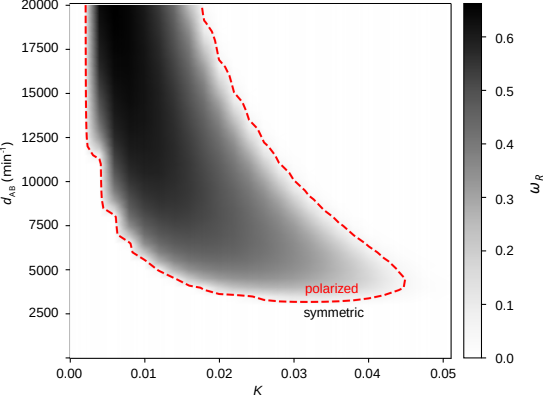


**Figure A3.** Estimated regions of instability in the *K*-*d*_AB_ plane. Plotted is the steady-state RomR polarity, ω_R_, for different combinations of the feedback parameters *K* and *d*_AB_. All other parameters were fixed at their values in Table S5. Dashed line indicates the estimated boundary between the polarized and symmetric regions.

**References**

1. Konopka MC, Sochacki KA, Bratton BP, Shkel IA, Record MT, Weisshaar JC. Cytoplasmic protein mobility in osmotically stressed *Escherichia coli*. J Bacteriol. 2009; 191: 231–37

2. Mullineaux CW, Nenninger A, Ray N, Robinson C. Diffusion of green fluorescent protein in three cell environments in *Escherichia coli*. J Bacteriol. 2006;188: 3442-8.

3. Guzzo M, Murray SM, Martineau E, Lhospice S, Baronian G, My L, et al. A gated relaxation oscillator mediated by FrzX controls morphogenetic movements in *Myxococcus xanthus*. Nat Microbiol. 2018;3:948-59.

4. Nocedal J, Wright SJ. Numerical Optimization. Springer New York; 2006.

5. Virtanen P, Gommers R, Oliphant TE, Haberland M, Reddy T, Cournapeau D, et al. SciPy 1.0: fundamental algorithms for scientific computing in Python. Nat Methods. 2020;17:261-72
