# Supplementary material for: Protein-protein interaction network controlling establishment and maintenance of switchable cell polarity
Source: PLoS Genet. 2020 Jun 22;16(6):e1008877. doi: 10.1371/journal.pgen.1008877 (PMC7332107; doi:10.1371/journal.pgen.1008877)
Supplement: Attachment — Submitted filename: Carreira_Rebuttal.docx [file pgen.1008877.s020.docx]

This study presents a logical and interesting set of experiments to characterize a bacterial cell polarity circuit. The experiments appear to be well executed and quantified, the inferred interaction topology is well supported, and the model provides additional insight with several quite simple assumptions. With the correction of the minor errors below, I recommend acceptance.

Fig. 2A,B-some labels on the bottom panels appear to be incorrect: A) mutant label should say deltaBdeltaR? and B) wild-type label should say A+R+?
**Response:** We apologize for the mistake & have corrected the errors. Moreover, we re-checked all figures for correct labeling.

Reviewer's Responses to Questions
**Reviewer #1:** This study presents a systematic analysis of the cell polarity underlying motility in M. xanthus. By studying polarity in fluorescence microscopy in all relevant strain backgrounds and doing a quantitative analysis (importantly distinguishing polar localization and asymmetry between poles as well as polar cluster intensity and fraction of cells with polar clusters), the authors very systematically dissect the regulatory networks underlying polarity. The results are then incorporated into a mathematical model to obtain further insight.

In my opinion, this is a exemplarily beautiful study and this is one of the rare cases where a paper could be accepted without revisions. The only point that remains open is the dynamics of switching, which in the “basic” scenario with only RomR does not happen, while it is typical for the WT. However, this is likely beyond the current study, but might be interesting to address in a follow-up study.
**Response**: Thank you very much. We agree with the reviewer that the question remains about the dynamics of switching. In our future research, we will address exactly this question.

**Reviewer #2:** This is an interesting paper that addresses the polarity switching in a bacterium, combining experiments that quantify the abundance of singling molecules at the cell poles with a mathematical model to describe the dynamics.

Since my primary expertise is in eukaryotic cell polarity modelling, I have little to say about the specific biological details presented here, other than the comment that I found it interesting to see the similarities (as well as differences). I will mainly comment about the mathematical model and some suggestions for increasing clarity and readability.

Overall, I believe that the paper is of interest and merits publication.

Signed: Leah Edelstein-Keshet

Major Comments:
The authors are using a modified version of a model previously presented elsewhere, so this explains why the description of the model is brief. That said, the paper should be self-contained, so a little more detail would be useful, primarily in some text in a Supporting Information section. (See specific suggestions below). The authors mention some analysis (e.g. linear stability), and some of those details - even abbreviated - would be helpful in the Appendix. The first 2 equations of the model are a bit like species-competition equations, with R modulating the relative activation rates of the competitors. Such equations are relatively easy to analyze, and the parameter dependence can be easily visualized in bifurcation diagrams. In particular, the regimes of instability could be quantified fairly easily, I believe.
**Response**: We appreciate the comments of the reviewer. In the revised manuscript, we have added a new Supplementary Appendix in which we present these aspects of the model in more detail, including linear stability analysis of the mutant conditions and different stability regimes of the WT models. We refer to the Supplementary Appendix in the description of the model on page 24-26. Because this text is quite long (11 pages), we decided to include the more detailed description of the model incl. the linear stability analysis as an appendix rather than in the Methods section.
We have also included, as a new supplementary figure (Fig. S9A), a bifurcation diagram showing the stability regimes as a function of the MglA feedback parameter K which, as was previously stated in the Discussion, we find to be the key parameter that must be tuned to achieve bistability. Additionally, in the new Fig. S9B, we show the regime of instability as a function of K and the MglB-MglA regulation rate d_AB_. We refer to the new figure on p. 18 para. 2.

For the simulations, the authors used some custom programs, but since the ODEs are so very standard, they could be easily handled by commonly available software, e.g. XPP, MatLab, Morpheus. (In XPP bifurcation analysis would be easy to do. In Morpheus, the ODEs could also be extended to a bacterial-shaped elliptical domain.) An advantage of this is also that the code is easily shared with others or uploaded as as SI, so students can play with the model. It would make the paper much more fun to read and experience. I realize that some of this is perhaps a bit beyond the purpose of the current study (e.g. the spatial version), but I recommend it as a relatively easy way to go to the next step.
**Response**: We have made the source code for the programs publicly available at <https://github.com/gerland-group/mxanthus_polarity> (this URL is also now included in the text on p. 26). While we appreciate that the equations are largely standard, we do not believe that repeating the study using alternative software would provide a significant benefit. In particular, given that the protein localization appears to be highly confined to the cell poles, and that we do not know many of the molecular details of polar localization (e.g. the nature and configuration of polar targeting sites, extent of membrane diffusion), we think (and we hope that you agree with us) there is little value in developing a model that explicitly builds in the cell geometry.

The models are all two-compartment ODEs (with conservation laws for the cytoplasmic compartment). This resembles the treatment of Rac-Rho polarity in melanoma cells in the pair of papers:

Holmes, William R., et al. "A mathematical model coupling polarity signaling to cell adhesion explains diverse cell migration patterns." PLoS computational biology 13.5 (2017): e1005524.

Park, JinSeok, et al. "Mechanochemical feedback underlies coexistence of qualitatively distinct cell polarity patterns within diverse cell populations." Proceedings of the National Academy of Sciences 114.28 (2017): E5750-E5759.

In that case, feedback from the extracellular matrix (ECM) affected the competition, replacing the role of the R in the model by the authors. One takeaway from that pair of papers was that it can be helpful to go through a couple of the "failed models" to show what is essential and what is not. (This could be done in the SI, and would make the paper more attractive to modellers.)
**Response:** We thank the reviewer for drawing our attention to these papers, and we now comment on the mechanistic similarity in the Discussion p. 20 para 1.
In the Supplementary Appendix, we now include the stability analysis that shows that the interactions of all three proteins is essential for instability. We also further discuss the variant models, including providing phase diagrams indicating the combinations of the feedback parameters for which each model becomes unstable. We also emphasize why we consider the model in which MglA acts only directly on MglB to be inconsistent with our experimental data.

The authors say nothing about diffusion across the cell. It would be helpful to get an estimate of the diffusion times, and how they compare with the other kinetic timescales. I suspect that it is entirely reasonable to ignore diffusion in these small cells, but a brief reasoning in the SI is justified. I liked the description of parameter estimation and hope this could be detailed further in the SI, as it is an important aspect of the model realism.
**Response:** We have included in the Supplementary Appendix some discussion on the validity of the assumption that the cytoplasmic pool of proteins can be considered well-mixed.
We also present in the Supplementary Appendix a more detailed description of how the parameter values were determined including how the mutants were employed and the fitting algorithm. The corresponding section in the Methods of the main text has been slightly reduced to limit repetition.

Minor issues

Circles in Fig 1A: represent molecules as well as amounts.. possibly confusing. The caption does not help explain this, and the font size on the lower part of Fig1A is too small.
**Response:** We apologize for the confusion. We now state in the caption to Fig. 1 that “Amounts of each proteins localized at each pole are indicated by the size of the corresponding cluster.”

Add the definitions of A, B, R to some figs such as Figs 2, 5, 6
**Response:** Good point! We now state in the caption to Fig. 2 that “Here and in the following figures, *A*^+^, *B*^+^ and *R*^+^ are short for *mglA^+^*, *mglB*^+^ and *romR*^+^; Δ*A,* ∆*B* and ∆*R* are short for ∆*mglA*, ∆*mglB* and ∆*romR*, respectively”.

In Description of mathematical model. (L 745-759) you can easily add the definitions of the variables, e.g.

cellular pools that represent the fraction of each protein that is localized at each of the two cell poles, and the delocalized fraction.

-->

cellular pools that represent the fraction of each protein that is localized at each of the two cell poles (A_i, B_i, R_i for i=1,2) , and the delocalized fraction (A_c, B_c, R_c).

Also include this in Fig 6 caption for better readability, and define A,B, R as levels of given components. Explain in caption for E that the size of the balls represents the amount of given substance that is localized at a pole.

**Response:** Good points! We have changed the text and captions as suggested to include this information.

L795

With the dissociation rates are fixed in this way

-->

When the dissociation rates are fixed in this way
**Response**: Thanks! This section of the text has been altered to reflect the new content in the Supplementary Appendix.
